# Improving seed germination and physiological characteristics of maize seedlings under osmotic stress through potassium nano-silicate treatment

**DOI:** 10.3389/fpls.2023.1274396

**Published:** 2023-12-20

**Authors:** Weria Weisany, Javad Razmi, Danial Pashang

**Affiliations:** ^1^ Department of Agriculture and Food Science, Science and Research Branch, Islamic Azad University, Tehran, Iran; ^2^ Department of Plant Protection, Science and Research Branch, Islamic Azad University, Tehran, Iran

**Keywords:** osmotic potential, potassium nano-silicate, seed germination, maize, nanotecehnology

## Abstract

**Introduction:**

Osmotic stress can significantly affect the survival and functioning of living organisms, particularly during vulnerable stages such as seed germination and seedling growth. To address this issue, advanced technologies like nanofertilizers have been developed to improve soil conditions and enhance plant growth in stressed ecosystems due to their multiple effects and efficient consumption.

**Methods:**

The objective of this study was to investigate the impact of potassium nano-silicate (PNS) on the physiological characteristics of maize seedlings and seed germination under various levels of osmotic stress induced by polyethylene glycol (PEG). The study considered two factors: two levels of PNS concentration (500 and 1000 ppm) and PEG-6000 solution with different osmotic stress levels (-2, -4, -6, and -8 bars).

**Results and discussion:**

The results demonstrated that the application of PNS at a concentration of 1000 ppm led to increased radicle length and hypocotyl length as well as fresh weight of maize seedlings. Furthermore, PNS at a concentration of 1000 ppm had a more beneficial effect on the germination rate of maize seedlings under osmotic stress compared to 500 ppm. Additionally, the application of PNS under osmotic stress conditions resulted in an increase in various physiological parameters, including protein content, chlorophyll a, chlorophyll b, total chlorophyll content, proline content, and the activity of catalase (CAT) and ascorbate peroxidase (AXPO) enzymes. These findings indicate that the use of PNS can have a positive impact on the physiological characteristics of maize seedlings and seed germination under osmotic stress conditions. Overall, this technology has the potential to enhance crop growth and yield in stressed ecosystems. By improving the survival and function of plants during vulnerable stages, such as seed germination and seedling growth, the application of PNS can contribute to more resilient agricultural practices and promote sustainable food production in challenging environments.

## 1 Introduction

Among various plant growth stages, seed germination and seedling growth are notably vulnerable to the detrimental impacts of drought stress (Warwell and Shaw, 2019). Nevertheless, the use of potassium has demonstrated its potential to alleviate these effects through the enhancement of photosynthetic material transfer, stimulation of deeper root penetration, and reduction in water consumption (Zahoor et al., 2017; Huang et al., 2023). Nanofertilizers, emerging as modern technologies, have significant attention for their numerous benefits. These include enhanced efficiency, improved soil conditions, and increased resilience of ecosystems facing stress.

Drought is the main abiotic factor responsible for reducing agricultural productivity worldwide, impacting plant growth on approximately 45% of the total cultivated land (Chieb and Gachomo, 2023). Drought stress reduces the water content in plant tissues, leading to growth limitations and physiological and metabolic changes (Agaei et al, 2020). Additionally, the availability of nutrients in the soil undergoes substantial alterations under drought stress conditions (Agaei et al, 2020). As a result, effective management of nutrition conditions under stress becomes crucial for successful plant production (Zhang et al., 2020). Water scarcity is a significant limitation in dry and semi-dry regions, impacting agricultural production (Hejazi et al., 2023). Nutrient addition can influence stress resistance, either enhancing or reducing it, depending on water availability (Li et al., 2020). Among essential fertilizer elements, potassium stands out as the most crucial cation, selectively absorbed by plants, and playing a vital role in plant physiology and metabolism by activating plant enzymes (Sardans and Peñuelas, 2021).

Potassium (K) serves as a vital macronutrient essential for numerous physiological processes in plants, including nutrient and water uptake, nutrient transport, and overall growth. Its significance becomes especially pronounced in challenging environmental conditions (Zörb et al., 2014). While not classified as essential for higher plants, Silicon (Si) has been the subject of extensive research, revealing its positive impact on the growth of various crop species (Marschner, 2011). Research has demonstrated that Si plays a pivotal role in enhancing a plant’s resilience to environmental stresses, such as drought, by regulating water balance and optimizing photosynthetic activity (Ahmed et al., 2011; Rizwan et al., 2015; Kim et al., 2017; Wang et al., 2017).

Previous research has shown that the application of potassium silicate improves growth characteristics, pigment content, and essential nutrient absorption in plants under drought conditions (Moustafa, 2013). Similarly, Si application increases the relative water content of maize (*Zea mays*) under water-deficient conditions, indicating improved water retention in cells (Kaya et al., 2006). Si application has also been shown to stimulate antioxidant defense in Saccharum officinarum under water deficit conditions (Verma et al., 2020).

In recent years, there has been a remarkable increase in crop yields, especially in cereals, playing a pivotal role in meeting the world’s nutritional needs (Zahedi et al., 2020). The application of diverse nanoparticles (NPs) in agriculture offers a promising avenue to enhance tolerance to a variety of abiotic stress factors (Weisany and Khosropour, 2023). Advances in nanotechnology have resulted in improved plant resilience against a wide spectrum of environmental stressors, encompassing challenges like drought, salinity, and various types of infections (Weisany and Khosropour, 2023). Recent studies by Fan et al. (2022) and El-Ashry et al. (2022) highlight the significant potential of Si nanoparticles (SiNPs) in agriculture, as they demonstrate superior effectiveness in mitigating various abiotic stresses compared to bulk materials. Furthermore, SiNPs have shown positive impacts on the development of different plant species, including tall wheatgrass (*Agropyron elongatum* L.), tuberose (*Polianthes tuberosa* L.), and lemongrass (*Cymbopogon flexuosus* (Steud.) Wats) (Azimi et al., 2014; Karimian et al., 2021; Mukarram et al., 2021).

Maize (*Zea mays* L.) is a highly versatile emerging crop known for its wide adaptability across a range of agro-climatic conditions. The imperative of comprehending the impact of osmotic stress on seed germination and its potential consequences for crop yield underscores the demand for innovative remedies like PNS application. Nano-fertilizers, particularly with a focus on PNS, have emerged as promising tools for bolstering sustainable crop production through the amelioration of environmental stress and the augmentation of yield. Nevertheless, there remains a notable knowledge gap, necessitating a more profound exploration into the specific effects of PNS on maize seedlings facing osmotic stress induced by PEG. This paper’s novelty is underscored by its concentrated investigation into the influence of PNS on maize seedlings under the stress of osmotic conditions induced by PEG. While previous research has explored the use of nano-fertilizers for improving crop growth and stress tolerance, this study hones in on the unique context of maize seedling germination and development in the presence of osmotic stress, a crucial but relatively understudied area.

This study aims to bridge this knowledge gap and evaluate how PNS fertilizer influences both the morphology and physiology of maize seedlings under osmotic stress conditions. Its primary goal is to elucidate the advantages and practical applications of PNS in fortifying maize crop resilience and boosting productivity in challenging environments. Understanding how nanofertilizers can alleviate osmotic stress effects is pivotal for sustainable agriculture, particularly in water-scarce or drought-prone regions. By providing invaluable insights into physiological responses and morphological alterations, this research can pave the way for more effective crop management strategies. Ultimately, this investigation seeks to scrutinize the impact of PNS on maize seedlings and their germination under varying levels of osmotic stress induced by PEG. The objectives encompass appraising PNS’s impact on seedling growth, pinpointing the optimal PNS concentration, and exploring its influence on an array of physiological parameters. The study also delves into the potential of PNS to enhance crop growth and fortify resilience in the face of osmotic stress, fostering sustainable food production in demanding environments.

## 2 Material and methods

### 2.1 Plant material and treatments

The research was conducted at the laboratory complex of Razi University of Science and Research, experiments followed a two-way factorial design, comprising of potassium silicate nanoparticles (PNS) concentration (500 ppm and 1000 ppm) and the application of PEG-6000 solution (-2, -4, -6, and -8 bars). The study was carried out in the year 2022, with three replications performed to validate the findings. To induce osmotic stress, polyethylene glycol (PEG) was used, which has been widely employed as an osmotic agent in similar studies. A control group was included, where distilled water was applied instead of PEG to maintain optimal growth conditions.

The maize seeds (*Z. mays* var. indentata) were immersed in PNS solutions with concentrations of 500 ppm and 1000 ppm for a duration of one day. Subsequently, these primed seeds at the nano level were exposed to osmotic stress by submerging them in PEG-6000 solutions with varying osmotic potentials, specifically -2, -4, -6, and -8 bars. The germination room provided favorable conditions for seed germination and seedling development. A consistent average daily temperature of 28°C was maintained to promote germination and growth. Additionally, the lighting regime followed a photoperiod of 16 hours of light and 8 hours of darkness during the nighttime, ensuring an appropriate light cycle for the plants.

### 2.2 Synthesizing potassium silicate nanoparticles

**Figure 1:**
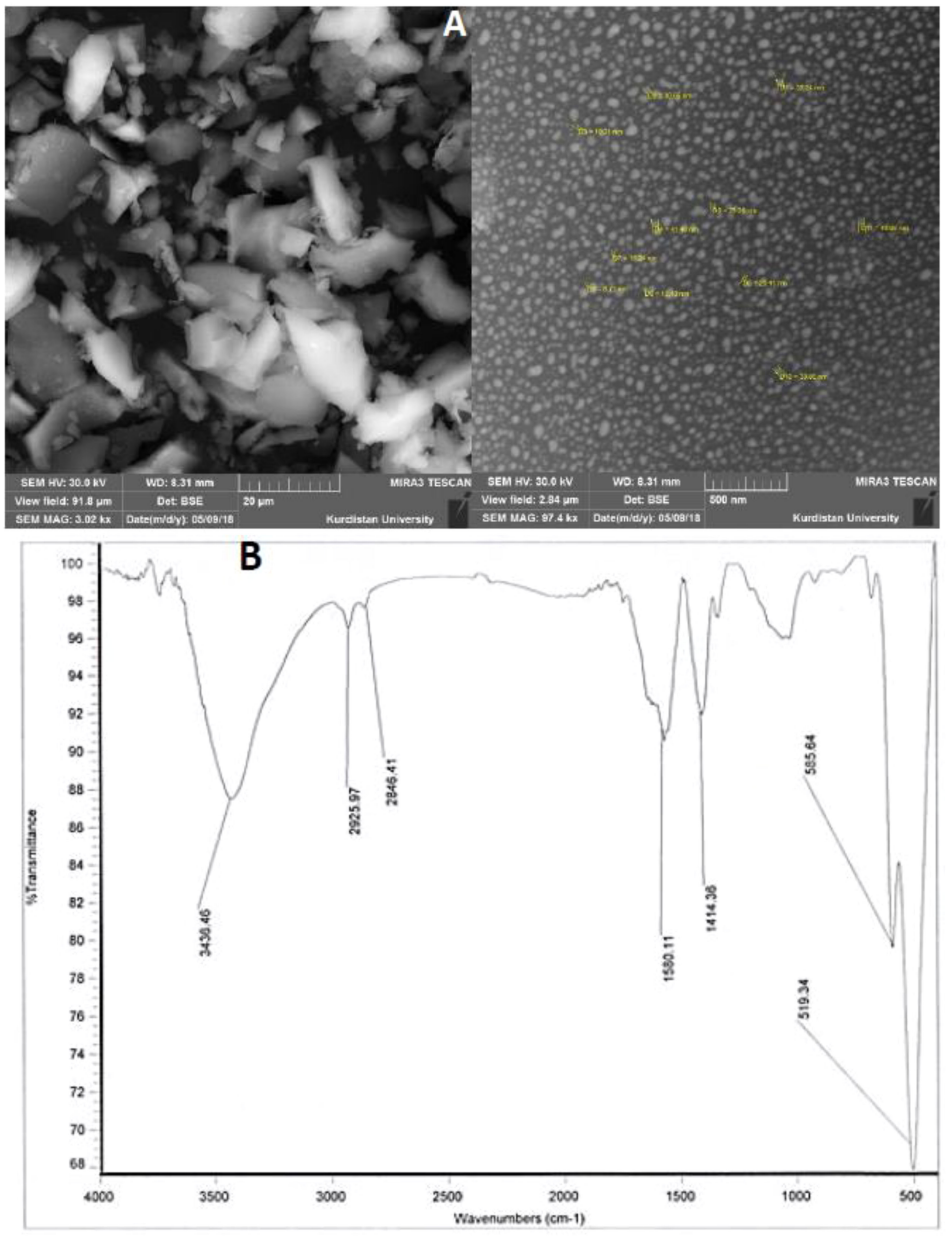
**(A)** Scanning electron micrograph (SEM) and **(B)** FT-IR spectra of potassium nano-silicate (PNS) nanoparticles.

The method described by Nandiyanto et al. (2017) was followed to synthesize potassium nano-silicate. Initially, rice straw was exposed to a high temperature of 973 K in a furnace, leading to the formation of rice straw ash. The resulting ash was finely ground and then combined with a potassium hydroxide solution. The mixture underwent stirring at 900 rpm while being heated at 338K for 2 hours. Subsequently, the extraction solution was introduced into a flame-assisted spray-pyrolysis apparatus, where droplets were introduced into the flame apparatus using a controlled airflow and commercial liquid petroleum gas (LPG; Pertamina). The synthesized PNS were subjected to characterization through Scanning Electron Microscopy (SEM) and Fourier-Transform Infrared Spectroscopy (FT-IR) analysis. **Figure 1** displays a representative image. The results obtained from SEM and FT-IR analyses effectively elucidated the structural features of the PNS. To prevent the aggregation of PNS, the pH was lowered to approximately 3, which closely approximated the conditions of CO_2_ saturation (Kim et al., 2015).

### 2.3 Germination percentage and morphological traits

The cultivation method for the germination study involving maize seeds was meticulously executed as follows:

Germination paper, specifically designed for germination studies, was positioned on a petri dish with a snug-fitting lid to prevent any moisture loss. This germination paper was then meticulously saturated with water, creating an optimal environment to facilitate seed germination. Three rows of maize seeds, each containing 50 seeds, were gently and precisely positioned in the center of the germination paper. This careful arrangement ensured even distribution of seeds for the study. The experiment adhered to a controlled light and temperature schedule, simulating optimal conditions for maize seed germination. It consisted of a 16-hour period of light at 28°C, mimicking daylight conditions, followed by 8 hours of darkness at 25 ± 1°C, resembling nighttime conditions. Throughout the experiment, regular visits were made to monitor the germination progress of the seeds, with the number of germinated seeds observed and recorded daily. Seed germination percentage was calculated from the first seed’s germination until the final day of the germination process, using the equation developed by Vollmann and Eynck (2015).

During the seed germination phase, we introduced 10 mL of each treatment solution into separate petri dishes while ensuring that the filter paper maintained its moisture. This procedure was conducted with three replicates for each treatment. After 20 days of cultivation, we randomly selected ten healthy seedlings and measured their radicle (root) length and hypocotyl (stem) length with a ruler accurate to one millimeter (mm). These measurements yielded valuable insights into the early growth characteristics of the seedlings. At the end of the germination period, we harvested the plants from each petri dish. To assess overall plant development, we measured their fresh weight using a sensitive balance and meticulously recorded the corresponding weights for further analysis and comparison. This comprehensive cultivation method provided strict control and systematic data collection, enabling a thorough examination of maize seed germination and early growth characteristics.


GP= (Total number germinated seeds/total number seeds) *100

### 2.4 Soluble protein content

After harvesting the ten seedlings, they were meticulously crushed until they transformed into a fine powder. Subsequently, 0.5 g of the powdered leaf samples were transferred to 2-mL microtubes, and 1 mL of extraction buffer (phosphate buffer with pH=7) was added. The mixture was vigorously vortexed (Variable Speed Vortex Mixer; USA) for 30 seconds. Afterward, the microtubes were centrifuged (Eppendorf 5417R Centrifuge; Canada) at 14,000 rpm for 15 minutes at a temperature of 4°C. Upon completion of the centrifugation, the resulting supernatants were carefully collected using a pipette and transferred to 1.5-mL microtubes. They underwent a second centrifugation at 14,000 rpm for 10 minutes at °C. Following the second centrifugation, the supernatants were collected using a pipette and transferred to microtubes of the same volume. Throughout the process, the microtubes containing the extracts were kept on ice. If not immediately used, the microtubes were transferred to a -80°C freezer for storage (Beauchamp and Fridovich, 1971). These extracts were utilized to measure the protein content of the total solution and the enzymes. The quantification of total soluble protein content was carried out in accordance with the method established by Bradford (1976), with bovine serum albumin (BSA, Sigma Aldrich) employed as the designated standard.

### 2.5 Enzyme assay

The activity of ascorbate peroxidase (APX, EC 1.11.1.11) was determined following the method described by Nakano and Asada (1981). In a final volume of 3 mL, the reaction mixture consisted of 50 mM potassium phosphate buffer (pH 7.0), 0.2 mM EDTA, 0.5 mM ascorbic acid, 2% H_2_O_2_, and 0.1 mL of enzyme extract. The absorbance at 290 nm was measured for 1 minute to track the decrease, and the amount of ascorbate oxidized was calculated using the extinction coefficient (ε = 2.8 mM^-1^ cm^-1^). APX activity was defined as the oxidation of 1 mmol of ascorbate per minute per milliliter of the reaction mixture at 25°C. Catalase CAT (EC 1.11.1.6) activity was assessed following the methodology of Weisany et al. (2012) with some minor adjustments. To measure the activity, a reaction mixture (1.5 mL) was prepared, comprising 100 mmol L^-1^ phosphate buffer (pH 7.0), 0.1 mmol L^-1^ EDTA, 20 mmol L^-1^ H_2_O_2_, and 20 µL of enzyme extract. The reaction was initiated by adding the extract, and the reduction of H_2_O_2_ was monitored at 240 nm. The quantification was based on the molar extinction coefficient (36 M^-1^ cm^-1^), and the results were expressed as CAT units per milligram of protein (U = 1 mM of H_2_O_2_ reduction per minute per milligram of protein).

### 2.6 Proline content

Proline measurement was performed following the method described by Bates et al. (1973). Leaf tissue weighing 0.5 g was ground in a mortar and homogenized in 10 mL of 3% sulfosalicylic acid. The resulting extract was then clarified. In a test tube, 2 mL of acetic acid and 2 mL of ninhydrin solution were added to 2 mL of the clarified extract. The test tubes were heated in a water bath at 100°C for one hour and then cooled on ice. To dissolve proline in toluene, 4 mL of toluene was added to each test tube, and vigorous shaking was performed to separate the two distinct layers: a red-colored toluene layer (upper layer) containing proline and a colorless layer (lower layer) without toluene. The proline content in the toluene layer was determined by measuring the absorbance at 520 nm using a spectrophotometer (UV spectrophotometer UV1600, Angstrom Advanced Inc. USA - Massachusetts) and referring to the proline standard curve. The ninhydrin solution was prepared by dissolving 2 g of ninhydrin in 48 mL of acetic acid and 32 mL of 6 M orthophosphoric acid.

### 2.7 Chlorophyll content

Extraction of chlorophyll was performed using acetone, and its measurement was carried out using the modified method of Jeffrey and Humphrey (1975). For this purpose, during the vegetative growth stage and 20 days after applying osmotic stress and PNS treatments, 1 gram of each leaf sample was homogenized in 5 mL of 90% ethyl acetate. The obtained extract was then clarified, and its volume was adjusted to 10 mL by adding more ethyl acetate. Subsequently, the light absorption by the resulting extract was determined using a spectrophotometer at wavelengths of 663 nm and 645 nm. The concentrations of chlorophyll a, chlorophyll b, and their total were calculated using the following equations.


$$\text{Chlorophyll a content }\left(\text{mg m}{\text{L}}^{-1}\right)\;=\;12.7\;\left(\text{A }663\text{ nm}\right)\;-\;2.69\;\left(\text{A}645\text{ nm}\right)$$

$$\text{Chlorophyll b content }\left(\text{mg m}{\text{L}}^{-1}\right)\;=\;22.9\;\left(\text{A }645\text{ nm}\right)\;-\;4.69\;\left(\text{A }663\text{ nm}\right)$$

$$\text{Chlorophyll a}+\text{b content }\left(\text{mg m}{\text{L}}^{-1}\right)\;=\;20.2\;\left(\text{A }645\text{ nm}\right)\;-\;8.03\;\left(\text{A }663\text{ nm}\right)$$

### 2.8 Data analysis

Data analysis was performed using SAS statistical software, and graphical representations were generated using Microsoft Excel. Mean comparisons were conducted using Duncan’s Multiple Range test with a significance level of 5%. The experiment was structured as a factorial experimental study following a randomized complete design (RCD) with three replications. The slicing technique was used to evaluate and compare the average interactions within the different treatments.

## 3 Result and discussion

### 3.1 Nanoparticle characterization

**Figures 1A, B** provide a visual representation of the SEM (with an average particle size of 25.4 nm) and FTIR spectra of the PNS nanostructured solution. The analysis was conducted on the powdered samples. In **Figure 1B**, the FTIR spectrum of PNS unveils distinct peaks at 3436.46, 2925.97, 2844.41, 1560.11, 1414.36, 585.64, and 519.34 cm^-1^. Notably, the FTIR spectra of the PNS nanostructured solution exhibit prominent peaks at 2925.97 cm^−1^ and 2844.41 cm^−1^, which can be attributed to the C–H stretching modes, encompassing both symmetrical and asymmetrical stretching of –CH_2_ and –CH_3_ functional groups (Parler et al., 2001). Furthermore, the spectrum of the PNS sample showcases peaks at 1560.11 and 1414.36 cm^−1^, signifying the incorporation of gentamicin into the silica matrix (Nampi et al., 2012).

### 3.2 Morphological traits

**Figure 2:**
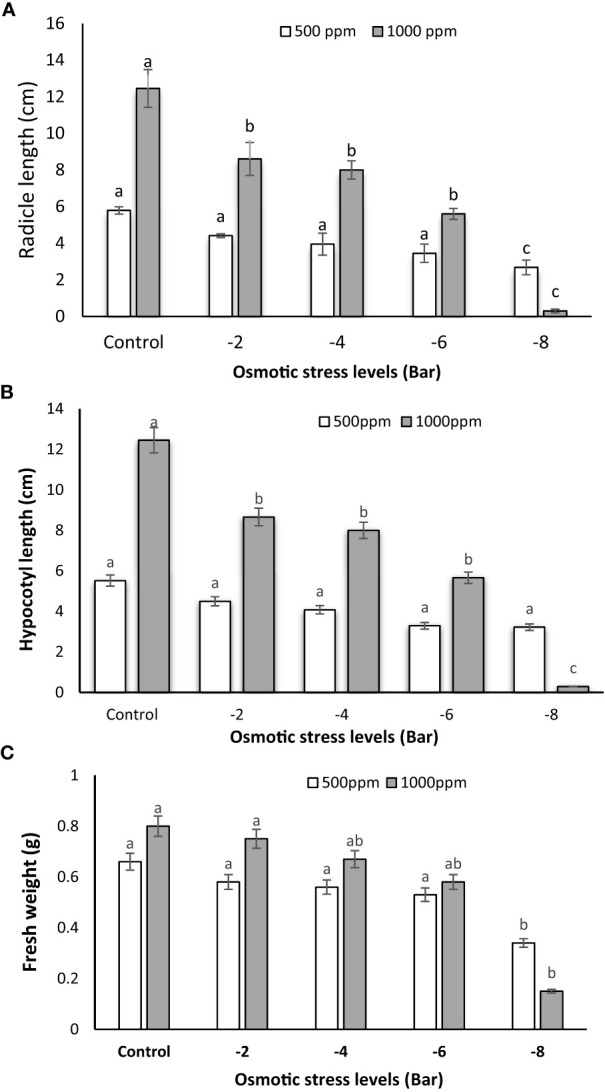
Radicle length **(A)**, hypocotyl length **(B)** and fresh weight **(C)** of maize seedlings subjected to different osmotic stress levels (-2, -4, -6, -8 bars) and two levels of potassium nano-silicate (PNS) concentration (500 and 1000 ppm). The treatments with at least one similar letter do not have a significant statistical difference on the probability level of 5%.

The application of 1000 ppm PNS resulted in a notable increase in radicle length as compared to the 500 ppm group, evident across various osmotic stress levels. (**Figure 2A**). This suggests that the higher concentration of PNS (1000 ppm) had a positive impact on radicle length, except under the -8 bar stress condition. According to **Figure 2C**, -8 bar osmotic stress caused a decrease in the fresh weight. However, the lower concentration of PNS (500 ppm) did not show a significant effect on radicle length, hypocotyl length, and fresh weight under any of the tested osmotic stress levels (**Figures 2A–C**). Similarly, when examining the average effects of PNS at 500 and 1000 ppm on hypocotyl length across various osmotic stress levels, a consistent trend emerges. Hypocotyl length exhibits a noticeable increase as the PNS concentration rises to 1000 ppm under osmotic stress levels of -2, -4 and -6 bar, as well as in the control group (**Figure 2B**).

Furthermore, result indicated that the application of 100 ppm of PNS, when compared to 500 ppm, led to an increase in the fresh weight of maize seedlings under osmotic stress conditions (-2, -4 and -6 bar). However, no significant differences were observed between the 500 ppm and 1000 ppm treatments when comparing them to each other or to the control (**Figure 2C**).

Potassium plays a pivotal role in cell enlargement as a crucial component of the growth process, regulated through turgor pressure. Its concentration within the vacuole is intricately linked to hormonal regulation, making potassium an indispensable element in facilitating growth (Vattani et al., 2012). Therefore, the observed growth improvements in radicle length, hypocotyl length, and fresh weight in response to higher concentrations of PNS are consistent with expectations. A recent literature review by Mukarram et al. (2022) delved into the effects of Si nanoparticles (NPs) treatment on phytohormone production. The study found that the application of Si NPs had a notable impact, leading to an increase in the concentrations of crucial phytohormones in the plant roots. Additionally, the expression of genes regulated by these phytohormones was also influenced by Si NPs treatment, as observed in the study by Lang et al. (2019). The improvement in growth parameters such as radicle length, hypocotyl length, and fresh weight of seedling in response to higher concentrations of PNS could be attributed to the uptake of PNS into the root system. This uptake may trigger the production of hormones, particularly IAA, which plays a vital role in regulating root growth.

### 3.3 Germination percentage

**Figure 3:**
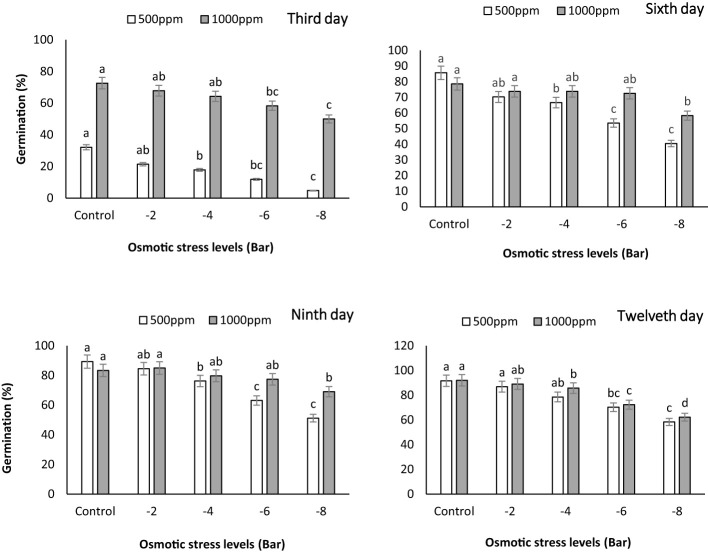
Germination percentage of maize seeds on the third **(A)**, sixth **(B)**, ninth **(C)** and twelfth **(D)** days subjected to different osmotic stress levels (-2, -4, -6, -8 bars) and two levels of potassium nano-silicate (PNS) concentration (500 and 1000 ppm). The treatments with at least one similar letter do not have a significant statistical difference on the probability level of 5%.

The germination percentage was measured on the third, sixth, ninth, and twelfth days during the growth period of maize seedlings. The graph reveals that, in most cases, the germination percentage was higher in the treatment with a PNS concentration of 1000 ppm (**Figure 3**). These findings indicate that PNS had a positive impact on the germination percentage of maize seedlings under osmotic stress. This result is in line with a study conducted by Sembada et al. (2023), in which they observed improved germination of tomato (*Solanum lycopersicum*) seeds when subjected to silica nanoparticles. They noted that smaller nanoparticles, as a general rule, exhibited greater effectiveness in promoting germination than their larger counterparts. This could be attributed to the increased contact area between the smaller particles and the seed’s surface compared to the larger particles. One possible explanation for the positive effect of chemical stimulants such as potassium nanoparticles on seed germination percentage is the establishment of hormonal balance in the seeds and the reduction of growth inhibitors like abscisic acid (ABA) and gibberellin (GA) (Mathur and Roy, 2020). The notable rise in germination percentage at the 1000 ppm concentration implies that using PNS can effectively improve the germination process, even in the presence of osmotic stress. This discovery underscores the potential of PNS as a valuable approach to enhance germination rates and support seedling establishment in water-scarce maize crops.

Si NPs have been found to promote seed germination in tomato plant, as demonstrated by Siddiqui and Al-Whaibi (2014). Furthermore, in a study conducted by Ghorbanpour et al. (2020), a remarkable increase of 27.3% was observed in shoot biomass. Moreover, there were substantial enhancements in pigment contents, with total chlorophyll levels increasing by up to 17.1% and carotenoid levels by up to 24.1% in drought-stressed barley (*Hordeum vulgare*) that had shown signs of recovery, thanks to the application of Si NPs at a concentration of 125 mg L^−1^. The germination stage plays a crucial role in plant development, requiring sufficient water availability to initiate vital metabolic processes. Our study revealed a noteworthy positive impact on the germination percentage of maize seedlings exposed to various osmotic stress levels when treated with PNS. Similarly, other studies have shown improved seed germination in cucumber (Alsaeedi et al., 2019) (200 mg L^−1^), maize (Karunakaran et al., 2016) (1000 mg L^−1^), through the use of Si NPs. Furthermore, Rahimi et al. (2021) demonstrated that priming marigold (*Calendula officinalis* L.) seeds with Si NPs (500 mg L^−1^) led to increased germination percentages, even under PEG-induced drought stress (0, −0.5, −1, and −1.5 MPa).

### 3.4 Soluble protein

**Figure 4:**
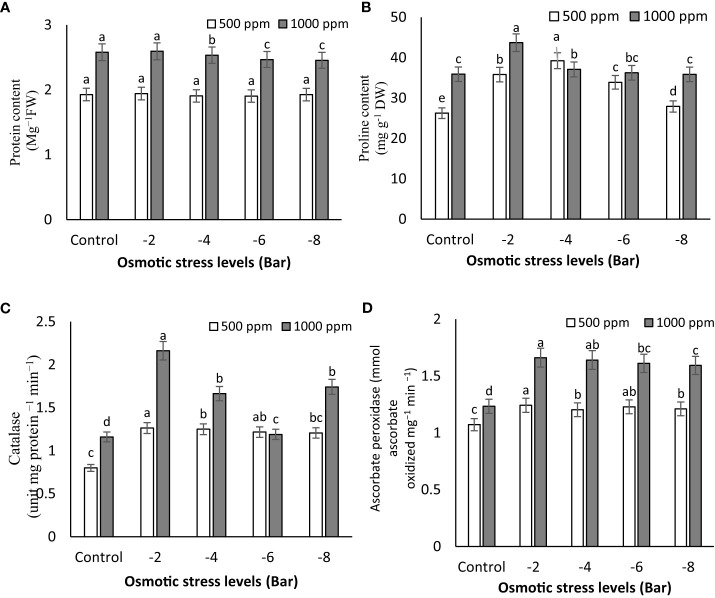
Soluble protein content **(A)**, proline content **(B)**, and the activity of catalase **(C)** and ascorbate peroxidase **(D)** enzymes of maize seedlings subjected to different osmotic stress levels (-2, -4, -6, -8 bars) and two levels of potassium nano-silicate (PNS) concentration (500 and 1000 ppm). The treatments with at least one similar letter do not have a significant statistical difference on the probability level of 5%.

The results presented in **Figure 4A** demonstrate a substantial increase in protein content in maize seedlings under control, -2, and -4 bar stress conditions when treated with a PNS concentration of 1000 ppm, in comparison to the 500 ppm treatment (**Figure 4A**). These results provide additional support for the concept that protein levels increase under moderate osmotic stress. Furthermore, the application of PNS at 1000 ppm to osmotic-stressed maize seedlings leads to a remarkable improvement in soluble protein content when compared to plants experiencing osmotic stress alone. Under osmotic stress conditions, there was no noteworthy difference in protein content between non-PNS seedlings and PNS-treated seedlings at a concentration of 500 ppm (**Figure 4A**).

Protein accumulation plays a crucial role in maintaining osmotic balance and membrane stability under stressful conditions, as emphasized in the research conducted by Qaseem et al. (2019). This finding strongly supports the notion that the higher concentration of PNS fertilizer has a notable positive impact on promoting protein synthesis in the seedlings. This observed increase can be attributed to the essential role of potassium in protein synthesis. It has been reported that potassium plays a crucial role in the final stages of protein production, facilitating its synthesis and transportation within the plant (Wang et al, 2013). Therefore, when potassium levels are insufficient, protein synthesis is hindered, leading to reduced protein content and an accumulation of amino acids and amides instead. Dørup and Clausen (1989) have noted that potassium deficiency results in an accumulation of hydrocarbon compounds in plants, indicating a delay in protein synthesis. Hence, ensuring an adequate supply of potassium is vital for promoting proper protein production in plants.

### 3.5 Proline content

**Figure 4B** illustrates the relationship between proline content and the severity of osmotic stress. It is evident that the proline levels showed a partial increase under osmotic stress conditions (-2 and -4 bar). Among the treatments, the plants exposed to -2 bar stress and treated with 1000 ppm PNS demonstrated the highest accumulation of proline (43.70 mg g^-1^ DW), while the control plants and those treated with 500 ppm PNS exhibited lower proline levels (26.25 mg g^-1^ DW) (**Figure 4B**). This finding aligns with previous reports on osmotic adjustment in chickpea (*Cicer arietinum*) under water deficit conditions (Gunes et al., 2008). The increase in proline content in leaves under water stress signifies an efficient mechanism for osmotic regulation, stabilization of sub-cellular structures, and cellular adaptation to limited water availability (Valentovic et al., 2006; Gunes et al., 2008). The application of PNS had an impact on proline accumulation in maize seedlings, emphasizing the significance of biochemical changes, such as alterations in protein, soluble sugar, proline, and chlorophyll levels, in plant responses to drought stress (Luo et al., 2023). The elevation of proline levels during water-limited conditions serves multiple functions, including acting as an energy sink to regulate redox potentials, scavenging hydroxyl radicals, protecting macromolecules against denaturation, and reducing cellular acidity (Smirnoff, 1993; Kishor et al., 1995). Furthermore, the rise in proline levels, influenced by hormonal regulation, plays a crucial role in improving the relative water content in leaves. These increased levels act as osmotic regulators, contributing to the plant’s enhanced tolerance to stressful conditions (Abd_Allah et al., 2017).

These results align with the study conducted by Siddiqui et al. (2015), demonstrating that plants treated with nano-silicon dioxide (6 g L^−1^) displayed improved proline content, photosynthetic rate, and water use efficiency in comparison to plants treated solely with salt and without nano-silicon dioxide.

Although research on the impact of PNS on osmolyte accumulation is limited, existing reports present conflicting information. However, it is suggested that the greater accumulation of proline in soybean plants due to silicon supplementation enhances tissue water content and counteracts the harmful effects of reactive oxygen species (ROS) induced by salt stress, thereby protecting the photosynthetic system (Mali and Aery, 2008). Additionally, the observed osmolyte accumulation in Si-supplemented plants may be attributed to the direct influence of Si on the metabolism and breakdown of vital osmotic components.

### 3.6 Catalase and Ascorbate peroxidase enzyme

Osmotic stress treatments influenced the activity of CAT, as shown in **Figure 4C**. With increasing of osmotic stress (-2 bar), CAT activity in maize seedling plants increased. The application of PNS had a significant impact on CAT activity. Notably, the seedlings treated with 1000 ppm of PNS demonstrated the highest CAT activity (2.16 mg protein ^−1^ min^−1^), particularly under the -2 bar osmotic stress level (**Figure 4C**). Enhanced activity of antioxidant enzymes, including CAT and APX, plays a crucial role in enhancing plant resilience to stress. It is well-established that alterations in antioxidant content represent one of the plant’s adaptive responses for regulating physiological and biochemical processes in the face of stressors (Rajput et al., 2021). The findings underscore the important role of potassium as a signaling element in stimulating various biochemical pathways, thereby leading to heightened activity of antioxidant enzymes such as catalase and ascorbate peroxidase.

**Figure 4D** clearly demonstrate the impact of PNS application on the activity of APX in maize seedlings. Specifically, the seedlings treated with 1000 ppm of PNS exhibited the highest APX activity, measuring 1.66 mmol ascorbate oxidized mg^−1^ min^−1^ under -2 bar osmotic stress. Additionally, the application of PNS (1000 ppm) significantly improved the activity of APX in maize seedlings subjected to osmotic stress, as observed in **Figure 4D**. Several environmental stresses, including water deficit, can trigger degenerative reactions in plants, resulting in the production of reactive oxygen species (ROS) and causing additional oxidative stress (Guo et al., 2023). In order to protect themselves, plants have the ability to synthesize antioxidants such as carotenoids, ascorbate, and glutathione, and enhance the activity of antioxidative enzymes like POD, CAT, and APX (Rajput et al., 2021). CAT, which is specifically found in peroxisomes, transforms H_2_O_2_ into water and molecular oxygen, while POD decomposes H_2_O_2_ by oxidizing co-substrates such as phenolic compounds and/or antioxidants (Hasanuzzaman et al., 2020). A recent study conducted by Hajizadeh et al. (2022) revealed that the use of Si nanoparticles (NPs) effectively enhances the tolerance of rose plants (*Rosa damascena* Mill.) to drought stress induced by PEG. The application of PNS resulted in increased CAT and APX activity (as shown in **Figures 4C, D**) while simultaneously reducing H_2_O_2_ concentration. These findings highlight the potential of PNS in alleviating the adverse effects of osmotic stress and enhancing stress tolerance in maize seedlings. Enhancing the activity of antioxidant enzymes like ascorbate peroxidase and catalase is a crucial factor in making plants more resilient to stress. Increased activity of antioxidants is part of a plant’s adaptive response to stress, and potassium acts as a signaling element to activate various biochemical pathways that boost the activity of these enzymes, including CAT and APX (Che et al., 2022). To enhance plant resilience to stress, it is essential to boost the activity of antioxidant enzymes such as CAT and APX. Activating these antioxidant enzymes plays a critical role in enhancing the plant’s ability to withstand and combat stress effectively.

### 3.7 Chlorophyll content

**Figure 5:**
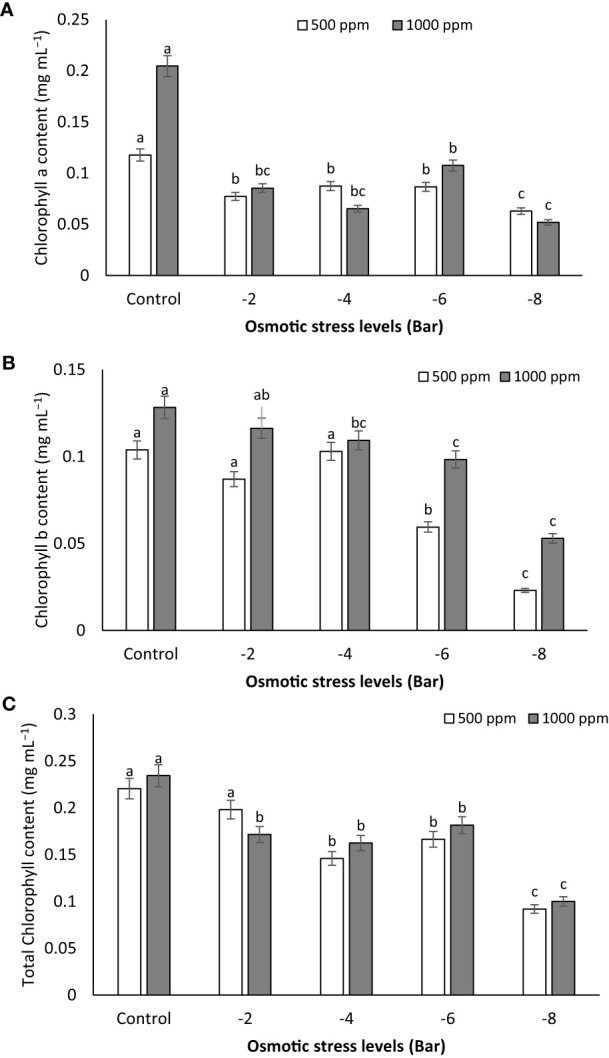
Chlorophyll a **(A)**, chlorophyll b **(B)** and total chlorophyll **(C)** content of maize seedlings subjected to different osmotic stress levels (-2, -4, -6, -8 bars) and two levels of potassium nano-silicate (PNS) concentration (500 and 1000 ppm). The treatments with at least one similar letter do not have a significant statistical difference on the probability level of 5%.

Observations indicate that osmotic stress exerts a notable influence on the reduction of chlorophyll a, b, and total chlorophyll levels (**Figures 5A–C**). In the control group, as the PNS concentration increased to 1000 ppm, the chlorophyll a (0.204 mg mL^-1^), b (0.128 mg mL^-1^) and a+b (0.234 mg mL^-1^) contents were the highest, whereas the -8 bar treatment exhibited the lowest values (**Figures 5A–C**). However, under conditions of osmotic stress (-2, -4 and -6 bar), there was no significant difference in chlorophyll a content between the 500 ppm and 1000 ppm concentrations (**Figure 5A**).

Drought stress amplifies the generation of reactive oxygen species (ROS), which inflict damage on plants by oxidizing photosynthetic pigments (Hasanuzzaman et al., 2020). In a study where water deficit was induced through PEG-8000 treatment (1, 2, and 3% polyethylene glycol), the application of 150 mg mL^-1^ Si NPs (50 nm) exhibited notable advantages for *in vitro* grown banana (*Musa acuminata* ‘Grand Nain’) shoots. It resulted in heightened growth, elevated total chlorophyll content, and mitigated electrolyte leakage (Mahmoud et al., 2020). Zahedi et al. (2020) have reported that the inclusion of Si NPs at a concentration of 100 mg mL^-1^ has the capability to enhance the levels of photosynthetic pigments and chlorophyll fluorescence in strawberry (*Fragaria × ananassa* Duch.) plants subjected to severe drought stress (25% FC). Similarly, the soil incorporation of nano-silicon at a concentration of 2 mM has been demonstrated to boost photosynthesis, chlorophyll levels, stomatal conductance, and transpiration in sugar beet (*Beta vulgaris* L. cv. Ekbatan) plants exposed to water deficit conditions (50% of crop evapotranspiration), as indicated by Namjoyan et al. (2020).

## 4 Conclusion

The utilization of nanoparticles, particularly PNS, emerges as a highly promising strategy to enhance plant drought tolerance and optimize crop production and management. Our research has illuminated the substantial benefits of applying PNS at concentrations of 500 and 1000 ppm. It has a positive influence on various parameters related to maize seedling germination and growth, whether in ideal conditions or when confronted with drought induced by PEG treatment. Notably, the most profound effects were observed with higher PNS concentrations (up to 1000 ppm), which significantly improved key physiological aspects such as protein content, chlorophyll levels (both a and b), total chlorophyll, proline content, and the activities of catalase (CAT) and ascorbate peroxidase (APX) enzymes. These findings underscore the efficacy of PNS application in enhancing physiological factors that contribute to sustainable or even enhanced plant growth during periods of drought stress.

## Data availability statement

The raw data supporting the conclusions of this article will be made available by the authors, without undue reservation.

## Author contributions

WW: Data curation, Methodology, Writing – original draft, Writing – review & editing, Project administration, Validation. JR: Writing – review & editing. DP: Data curation, Writing – review & editing.
