# Supplementary material for: Improving seed germination and physiological characteristics of maize seedlings under osmotic stress through potassium nano-silicate treatment
Source: Front Plant Sci. 2023 Dec 20;14:1274396. doi: 10.3389/fpls.2023.1274396 (PMC10765601; doi:10.3389/fpls.2023.1274396)
Supplement: Supplementary file 1 [file DataSheet_1.pdf]

Protein standard curve

The protein concentration was determined by employing a calibration curve that was established using the absorbance measurements of well-defined concentrations of Bovine Serum Albumin protein (BSA). The absorbance values of the protein samples of unknown concentration were subsequently matched against the BSA standard curve to ascertain their protein concentrations, following the method outlined by Bradford in 1976.

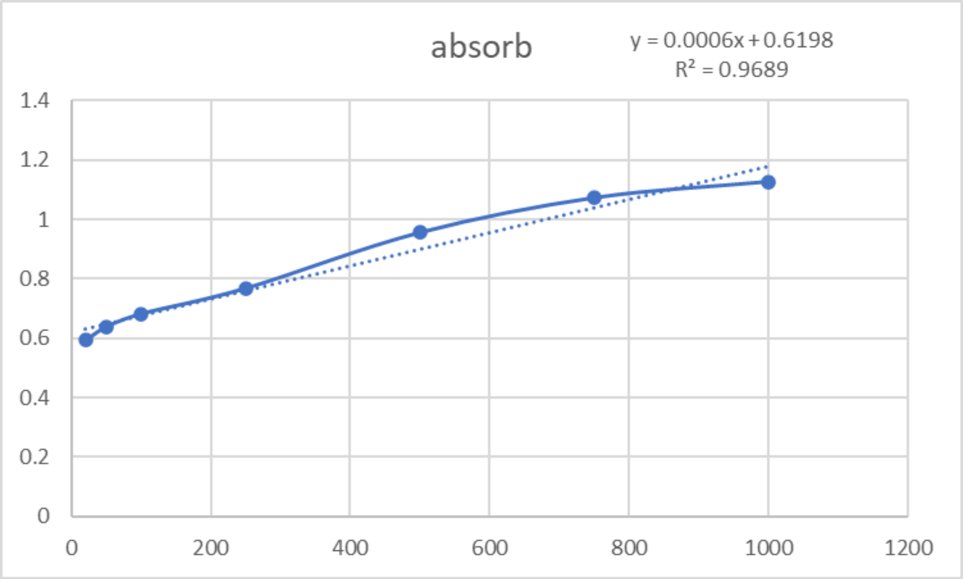

Proline standard curve

Preparation of the standard curve involved the addition of L-proline standard solutions, specifically 0.0, 0.20, 0.40, 0.60, 0.80, and 1.00 mL (corresponding to 0, 5, 10, 15, 20, and 25 µg of L-proline), into separate stoppered tubes. Each of these solutions was then diluted with water to reach a final volume of 1 mL. Subsequently, 0.25 mL of formic acid and 1.0 mL of ninhydrin-ethylene glycol monomethyl ether (EGME) solution were added to these tubes. The tubes were securely sealed with stoppers and subjected to boiling water for 15 minutes. They were then transferred to a 70°C thermostatic water bath for an additional 10 minutes. Following this, 15 mL of isopropanol was introduced, and after a 5-minute incubation period, absorbance readings were taken at 510 nm. The standard curve was constructed based on these measurements.

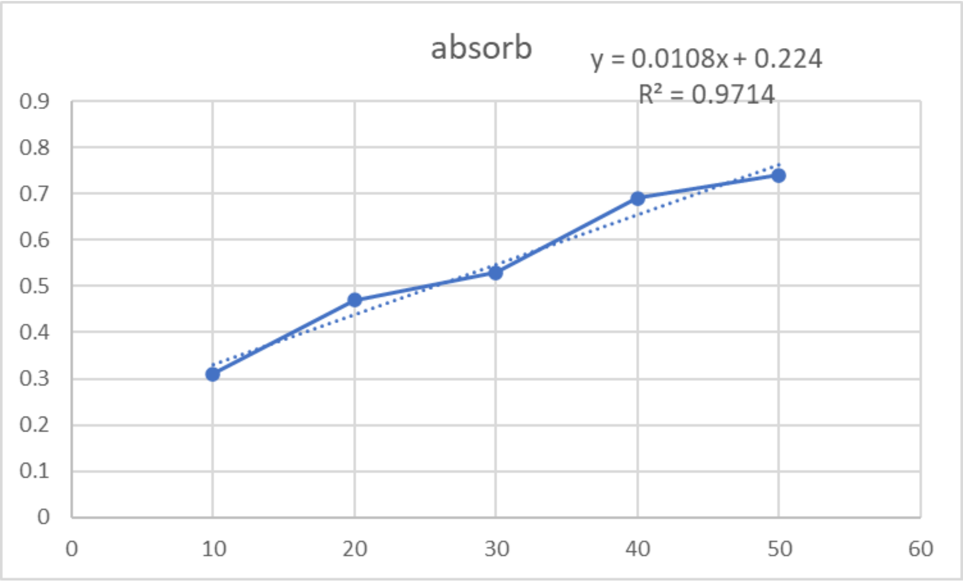
absorb
y = 0.0108x + 0.224
R² = 0.9714
0.9
0.8
0.7
0.6
0.5
0.4
0.3
0.2
0.1
0
0
10
20
30
40
50
60

Table 1: Variance Analysis of data from the measurement of radicle length, hypocotyl length, fresh weight, soluble protein content, proline content, catalase, ascorbate peroxidase, chlorophyll a, chlorophyll b and chlorophyll a+b of maize seedlings subjected to different osmotic stress levels (-2, -4, -6, -8 bars) and two levels of PNS concentration (500 ppm).

S.O.V	df	chlorophyll a+b	chlorophyll b	chlorophyll a	ascorbate peroxidase	catalase	proline content	Soluble protein content	fresh weight	hypocotyl length	radicle length
Osmotic stress (a)	4	0.03**	0.011**	0.011**	0.028**	0.800**	177.46**	0.001**	0.16 ns	10.76**	16.14ns
PNS (b)	1	0.06**	0.006**	0.006**	0.023**	0.169**	207.93**	0.038**	0.36 ns	0.20 ns	42.92 ns
a*b	4	0.003**	0.001**	0.000*	0.000**	0.010**	13.31**	0.000**	0.23 ns	24.90**	15.28 ns
Error	18	0.001	0.000	0.000	0.000	0.001	1.70	0.001	0.311	27.38	21.97

Ns (Not significant), * (significant at the 1% level), ** (significant at the 5% level), respectively, indicate the level of significance in the probability levels of 1% and 5%.

Table 2: Variance Analysis of data from the measurement of radicle length, hypocotyl length, fresh weight, soluble protein content, proline content, catalase, ascorbate peroxidase, chlorophyll a, chlorophyll b and chlorophyll a+b of maize seedlings subjected to different osmotic stress levels (-2, -4, -6, -8 bars) and two levels of potassium nano-silicate (PNS) concentration (1000 ppm).

S.O.V	df	chlorophyll a+b	chlorophyll b	chlorophyll a	ascorbate peroxidase	catalase	proline content	Soluble protein content	**fresh weight**	**hypocotyl length**	**radicle length**
Osmotic stress **(a)**	**4**	**0.04****	**0.016****	**0.03****	**0.18****	**25.86****	**67.86****	**0.02****	**0.79****	**65.13****	**240.98****
PNS **(b)**	**1**	**0.071****	**0.008****	**0.02***	**0.09****	**2.80****	**415.64****	**0.08****	**0.02 ns**	**1.58 ns**	**3.76 ns**
**a*b**	**4**	**0.004****	**0.000***	**0.01ns**	**0.00****	**2.73****	**5.23****	**0.006****	**0.16****	**23.21****	**75.42****
**Error**	**18**	**0.001**	**0.000**	**0.006**	**0.000**	**0.082**	**0.64**	**0.001**	**0.069**	**6.34**	**16.70**

Ns (Not significant), * (significant at the 1% level), ** (significant at the 5% level), respectively, indicate the level of significance in the probability levels of 1% and 5%.
